# The Leukocyte Receptor Complex in Chicken Is Characterized by Massive Expansion and Diversification of Immunoglobulin-Like Loci

**DOI:** 10.1371/journal.pgen.0020073

**Published:** 2006-05-12

**Authors:** Katja Laun, Penny Coggill, Sophie Palmer, Sarah Sims, Zemin Ning, Jiannis Ragoussis, Emanuela Volpi, Natalie Wilson, Stephan Beck, Andreas Ziegler, Armin Volz

**Affiliations:** 1 Institut für Immungenetik, Charité-Universitätsmedizin Berlin, Campus Virchow-Klinikum, Humboldt-Universität zu Berlin, Berlin, Germany; 2 Wellcome Trust Sanger Institute, Genome Campus, Hinxton, Cambridge, United Kingdom; 3 The Wellcome Trust Centre for Human Genetics, Oxford, United Kingdom; The Jackson Laboratory, United States of America

## Abstract

The innate and adaptive immune systems of vertebrates possess complementary, but intertwined functions within immune responses. Receptors of the mammalian innate immune system play an essential role in the detection of infected or transformed cells and are vital for the initiation and regulation of a full adaptive immune response. The genes for several of these receptors are clustered within the leukocyte receptor complex (LRC). The purpose of this study was to carry out a detailed analysis of the chicken *(Gallus gallus domesticus)* LRC. Bacterial artificial chromosomes containing genes related to mammalian leukocyte immunoglobulin-like receptors were identified in a chicken genomic library and shown to map to a single microchromosome. Sequencing revealed 103 chicken immunoglobulin-like receptor *(CHIR)* loci (22 inhibitory, 25 activating, 15 bifunctional, and 41 pseudogenes). A very complex splicing pattern was found using transcript analyses and seven hypervariable regions were detected in the external CHIR domains. Phylogenetic and genomic analysis showed that *CHIR* genes evolved mainly by block duplications from an ancestral inhibitory receptor locus, with transformation into activating receptors occurring more than once. Evolutionary selection pressure has led not only to an exceptional expansion of the CHIR cluster but also to a dramatic diversification of CHIR loci and haplotypes. This indicates that CHIRs have the potential to complement the adaptive immune system in fighting pathogens.

## Synopsis

The immune system developed to cope with a diverse array of pathogens, including infectious organisms. The detection of these pathogens by cells of the immune system is mediated by a large set of specific receptor proteins. Here the authors seek to understand how a particular subset of cell surface receptors of the domestic chicken, the chicken Ig-like receptors (CHIR), has evolved. They demonstrate that at least 103 such receptor loci are clustered on a single microchromosome and provide the first detailed analysis of this region. The sequences of the *CHIR* genes suggest the presence of inhibitory, activating, and bifunctional receptors, as well as numerous incomplete loci (pseudogenes) that appear to have evolved by duplications of an ancestral inhibitory receptor gene. Multiple regions of very high sequence variability were also identified within *CHIR* loci which, together with considerable expansion of the number of these genes, suggest that CHIR polypeptides are involved in critical functions in the immune system of the chicken.

## Introduction

Activating and inhibitory receptors containing domains of the immunoglobulin (Ig) superfamily are major components in regulating innate immunity of vertebrates [1,2]. These genes usually belong to multigene families containing several very similar members [3–5] arranged in tight genomic clusters [4–6]. Depending on their functions, the respective receptors can be grouped into three classes: (i) inhibitory receptors with a long cytoplasmic domain containing one or two immune receptor tyrosine-based inhibitory motifs (ITIMs) [7] or an immune receptor tyrosine-based switch motif (ITSM) [8], (ii) activating receptors with a transmembrane (TM) domain containing a positively charged residue which mediates association with immune receptor tyrosine-based activatory motif (ITAM)-containing adaptor molecules [9,10], and (iii) receptors like KIR2DL4 [11] and NCR2 [12] that combine activating and inhibitory features. The ratio of activating to inhibitory receptors varies widely between species. The human leukocyte immunoglobulin-like receptor (LILR) cluster, which is encoded within the leukocyte receptor complex (LRC), shows a balanced ratio of activating and inhibitory receptors [5], while the killer cell Ig-like receptor (KIR) cluster, which is also part of the LRC, reveals an haplotype-dependent, more or less pronounced excess of inhibitory receptors [3]. This is in contrast to the situation in the mouse, where the LRC seems to contain mainly activating paired Ig-like receptor (Pir) and no KIR genes at all [4].

Recently, it has been shown that activating KIRs are repeatedly lost during evolution and a significant amount of healthy individuals seems to be completely devoid of activating KIRs [13]. On the other hand, there must have been considerable evolutionary pressure to transform inhibitory ancestors into activating variants by co-opting ancient signaling pathways because such transformations occurred not only independently in several species but also within the structurally different but functionally similar killer cell lectin-like receptor subfamily A (Klra) genes of rodents [13]. For KIRs, this transformation dates back 13.5 to 18 million years ago, and especially the activating variants evolved extraordinarily fast [14]. This has already led to a high degree of nucleotide polymorphism, but the major changes result from exon shuffling [15], and involvement in pathogen defense has been suggested as a driving force [13]. Regarding all these aspects, the parallels between KIRs and activating Klras are striking [16,17].

KIR homologs are not known for the chicken, but chicken Ig-like receptors (CHIRs) were suspected to be LILR homologs [18] and they were initially identified by a database search with a mouse Pirb sequence [19]. However, the genomic organization of the CHIRs closely resembles the organization of most two-domain KIRs [17] and significant similarity has been demonstrated at the protein level. All ligands known so far for receptors with CHIR homology (e.g., KIR, LILR, Pir) are major histocompatibility complex (MHC) or MHC-related molecules. Some are even virus encoded [20] and mimic MHC expression on virus-infected cells. Many of these receptors are expressed by natural killer cells but they are present also in different combinations on subsets of myeloid cells and T lymphocytes [21]. Recently, expression and functionality of an inhibitory member of the CHIR gene family *(CHIR-B2, AJ879911)* have been demonstrated on B lymphocytes [22], and additional activating and bifunctional receptors and their expression were described [8]. In addition, a superficial analysis of the chicken LRC, using the publicly available sequence data, resulted in a preliminary estimate of the gene/pseudogene content of the cluster and a provisional phylogenetic analysis [23].

In this study, we present a comprehensive characterization of 550 kilobase-pairs (kbp) of the chicken LRC. The extremely high degree of variability on all levels, positive selection, independent development of activating and bifunctional receptors, as well as a tremendous gene expansion, suggest that CHIRs may be involved in the protection against highly variable pathogens.

## Results

### Library Screening

Screening of a chicken bursa of Fabricius (Bursa) cDNA library with a human *LILRA2* probe resulted in ten clones with high homology to the previously described *CHIRA* and *CHIRB* [19]. Sequence variation between these cDNAs suggested that they are derived from more than two genes. To clarify these findings, the Wageningen chicken genomic BAC library was screened with the CHIR-specific cDNA probes. The resulting 18 positive BAC clones could be mapped into four contigs by fingerprinting: contig 1: seven clones (12N4, 19H9, 52G8, 88M21, 93H17, 4C11, and 74P17), contig 2: three clones (35O24, 55M5, and 62I23), contig 3: two clones (7M1 and 104J15), and contig 4: two clones (58B13 and 112A23). Clones 7H19, 121G8, 121H22, and 126C12 could not be mapped to any contig and remained singletons.

Screening the BAC library with human LRC framework probes (see Materials and Methods) did not reveal additional clones. Analysis of zoo blots with the same framework probes resulted only in *LILRA2* hybridizing with chicken genomic DNA, reproducing earlier results [18].

### Chromosomal Localization

**Figure 1:**
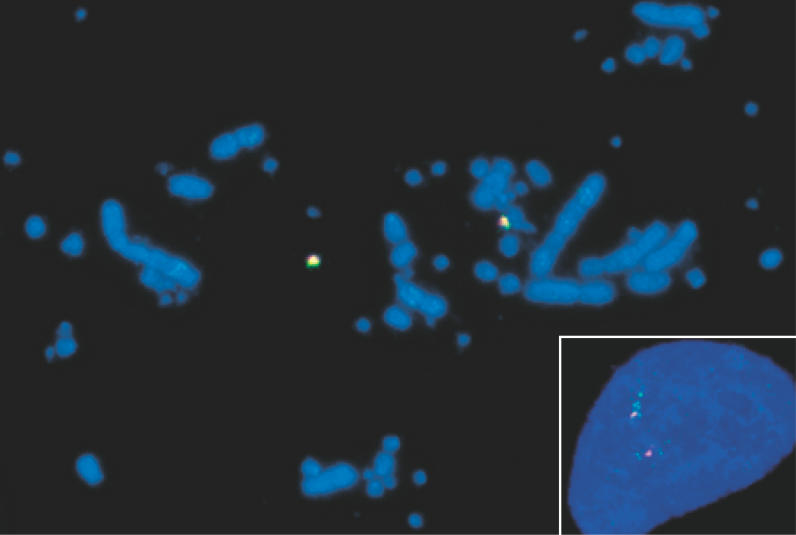
Chromosome Mapping of the Chicken LRC Dual-color FISH on chicken chromosome preparations showing colocalization of BACs 7H19 (red) and 93H17 (green) on metaphase (main picture) and interphase (inset). The clones map to a microchromosome, most likely belonging to group C (according to the chicken chromosome classification system proposed by [64]). The MHC-harboring BAC 65G09 did not colocalize to the same microchromosome (results not shown).

The chromosomal location of CHIR genes was determined using fluorescent in situ hybridization (FISH) to chicken chromosomes. The following BACs were mapped: 7M1, 7H19, 19H9, 52G8, 55M5, 62I23, 74P17, 93H17, 112A23, 121H22, and 126C12. Apart from 126C12, the BAC probes gave single well-defined signals on one microchromosome. Subsequent dual-color FISH in various combinations revealed colocalization of clones from different contigs on both, metaphase chromosomes and interphase nuclei (example in Figure 1). Colocalization was confirmed by FISH on DNA fibers (FiberFISH) (unpublished data). However, due to extensive cross-reactivity resulting from the high homology between CHIR genes and intergenic sequences, the relative position of contigs along the DNA fibers could not be established. Taking this strong cross-reactivity into account, we deduce that additional signals observed on other chromosomes with 126C12 are not CHIR derived.

### Genomic Organization

**Figure 2:**
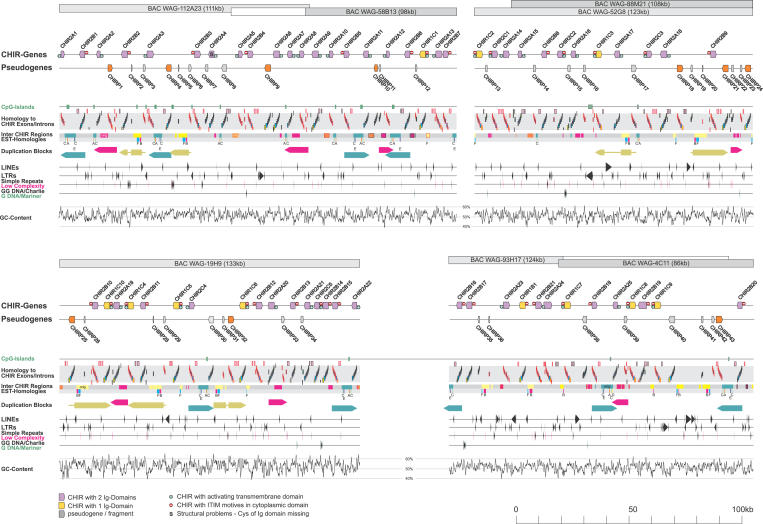
Genomic Organization of the Chicken LRC Gray boxes (top) represent the finished and submitted sequences of the corresponding BACs. White boxes represent the unfinished sequence overlaps between BACs that were analyzed but clipped to 2 kb as part of the submission process. All other tracks are labeled individually. Gene symbols are purple for CHIRs with two Ig-domains and yellow for one Ig-domain CHIRs, with the transcriptional orientation depicted by an arrowhead. Pseudogenes which were erroneously described by Nikoloaidis and colleagues [23] as functional are displayed in orange; all others are gray. Regions homologous to single CHIR exon/introns are presented as boxes with the following relation to the respective gray area: exon A above, exon B intersecting at the top, exon 1 at the top, exons 2 to 6 descending to exon 7 at the bottom, and exon C intersecting at the bottom. Intergenic regions labeled “Orig” were used to detect the respective homologous regions labeled by the same color. Duplication blocks are presented as arrows colored according to the enclosed intergenic regions. Note letters at the line “EST-homologies” and their specification in Table 3. A high-resolution version of Figure 2 is available as supporting material at http://www.charite.de/immungenetik/CHIR.

To determine the arrangement of CHIR genes, a total of seven BACs forming minimum tile paths of contig 1 (19H9, 52G8, 88M21, 93H17, and 4C11) and contig 4 (58B13 and 112A23) were selected for sequencing. The resulting sequence data confirmed an overlap of the two clones in contig 4 but failed to confirm contig 1, except for the overlap of BACs 93H17 and 4C11. As clone 88M21 turned out to lie entirely within clone 52G8 and since no overlaps between any of 52G8, 19H9, and 93H17 could be established, the CHIR gene cluster is thus currently represented by four stretches of sequence of unknown order and orientation (Figure 2). Further overlaps that could be used for gap closure do not exist as demonstrated by end-sequencing of the remaining clones. BACs of the presented contigs overlap to a considerable extent (Figure 2), but the overlapping material was clipped to 2,000 bp in the finished and submitted sequences. Using BLAST and dot matrix analysis, 103 regions with similarity to CHIR genes could be detected. 62 of the detected regions meet the minimal requirements for a functional CHIR protein (Figure 2), i.e., they code for a signal peptide, one or two Ig domains, and a TM domain. Regarding the nomenclature, an adaptation to the propositions of Viertlboeck and colleagues [22] (summarized in Table 1) is suggested, which has also been discussed with the poultry nomenclature committee. The remaining 41 regions consist of genes at different stages of decay and are predicted to be pseudogenes, but some may well be functional in other haplotypes or encode soluble CHIR proteins (details are provided in Table S1). Together, CHIR genes and pseudogenes cover up to 47% of the analyzed BACs (Table 2). In order to assess whether the contigs belong to two haplotypes, the arrangement of CHIR genes and noncoding elements, transcriptional orientation, and locus homology were compared between the contigs. No indications were found for an allelism, although the library employed here has been demonstrated to contain two haplotypes for several other loci (Dr. Crooijmans, Wageningen University, personal communication).

**Table 1:**
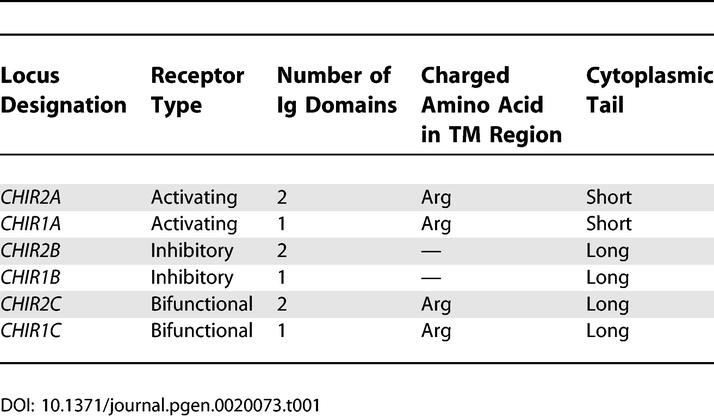
Nomenclature of CHIR Genes

**Table 2:**
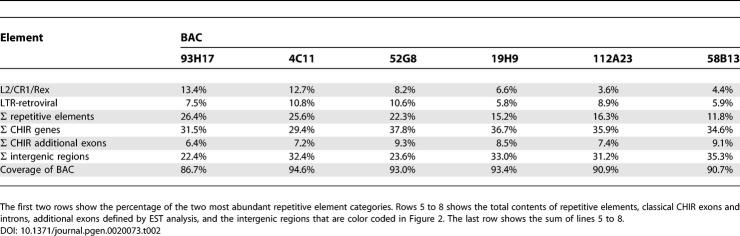
Content of CHIRs and Repetitive Elements of the Analyzed BAC Sequences

**Table 3:**
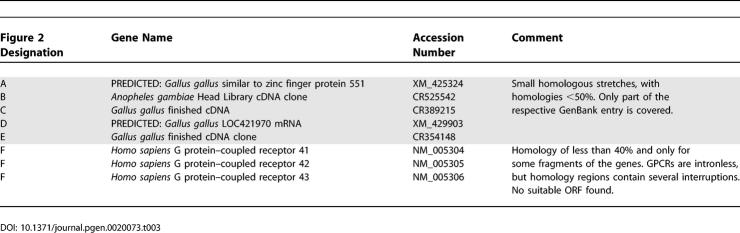
Summary of Expressed Sequences with Homology to Intergenic Regions of the CHIR Cluster

The locations of repetitive elements are shown in Figure 2, and a summary is provided in Table 2. The content of repetitive elements amounts up to 26.4% in the BACs and, together with the CHIR genes, leads to coverage of 50% to 60% (Table 2). Analysis of expressed sequence tags (ESTs) (see below) revealed three additional exons (A and B proximal of exon 1, C distal to exon 5), which have not been described so far. Especially exon A–/intron A–homologous sequences (Figure 2) are relatively frequent and, together with exons B and C, account for between 6.4% and 9.3% of the respective BACs. The remaining 30% to 45% of the BACs revealed significant genomic homology only to the BACs described here and resulted in the definition of four “inter-CHIR regions” (Figure 2) covering 22% to 33% of an individual BAC (Table 2). In total, these different elements encompass approximately 90% to 94% of the analyzed sequences (Table 2). The sequences still remaining are dispersed between the elements, in particular, upstream and downstream of the CHIR genes.

The analysis of the four inter-CHIR regions revealed that they are part of duplication blocks which include neighboring CHIRs (cf. seven copies of the “yellow” variant shown in Figure 2). Varying borders of these duplication blocks led in several cases to the duplication of incomplete CHIR genes (cf. CHIRP3, -P19, or -P29, Figure 2) and consequently to the generation of pseudogenes. From the association of all CHIR1C genes with the yellow intergenic region, additional smaller or decayed copies can be deduced. Further analysis of the intergenic regions revealed weak homologies with short discontinuous stretches of some genes and ESTs (Table 3), but these sequences do not reflect the structure of the respective genes, and homology with human LRC genes could not be demonstrated.

Of the 18 CpG islands that were predicted, four result from repetitive elements and one is part of the 14 copies of the intergenic region defined within BAC 4C11 (teal boxes, Figure 2). An association of these CpG islands with promoters of CHIR genes could not be established.

### Whole Genome Shotgun

During the analysis of the BAC sequences, the chicken genome consortium released the first draft sequence of the Red Jungle Fowl chicken [24], but the automatic assembly of the chicken LRC had not been successful. Nevertheless, to gain access to the Red Jungle Fowl LRC haplotype, we conducted a BAC-guided assembly including all available chicken whole genome shotgun (WGS) clones. In this new assembly, however, the only two CHIR gene containing contigs (13 and 57 kb) showed no significant homology to the BAC clone contigs presented here.

### Analysis of ESTs

**Figure 3:**
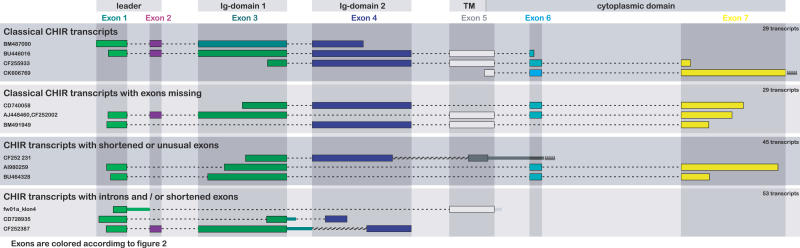
Examples of Alternative Splicing Found in CHIR Transcripts At the left are the accession numbers of the given examples. Spliced introns with correct splice donors and acceptors are displayed as single dotted lines. If splice sites do not meet the consensus sequences, introns are displayed as hatched lines. Introns not removed are presented as gray lines. The exon at the 3′ end of CF252231 shows only extremely weak homology to CHIR genes and was therefore colored gray. AAAAA indicates that the respective transcript contains a polyA tail.

Insight into the expression of CHIR genes was obtained through the analysis of 202 ESTs and cDNAs. The alignment (available as supplementary information at http://www.charite.de/immungenetik/CHIR), which could be obtained only by a semimanual strategy, led to a very complex picture, mainly due to extensive alternative splicing and variability of the genes. Since all exons, with the exception of exon 7, share the same reading frame [−1], the downstream exons are usually in frame. For the purpose of clarity, transcript variants were divided into six categories (Figure 3): (i) classical CHIR gene structure encompassing seven exons [22]; (ii) classical CHIR gene structure, but lacking between one and three exons. In all but one case, at least one Ig domain-coding exon was present; (iii) incomplete exons due to additional splice sites, with most of the respective intraexon 3 start positions being located in a hypervariable region; (iv) introns or parts of introns are present, partially combined with missing or truncated exons. Most of these sequences contain premature stop codons and do probably not code for functional receptors; (v) exon 7 replaced by a mariner transposon. These transcripts originate all from the Bursa library; (vi) presence of additional exons (A/B/C) in various combinations.

**Figure 4:**
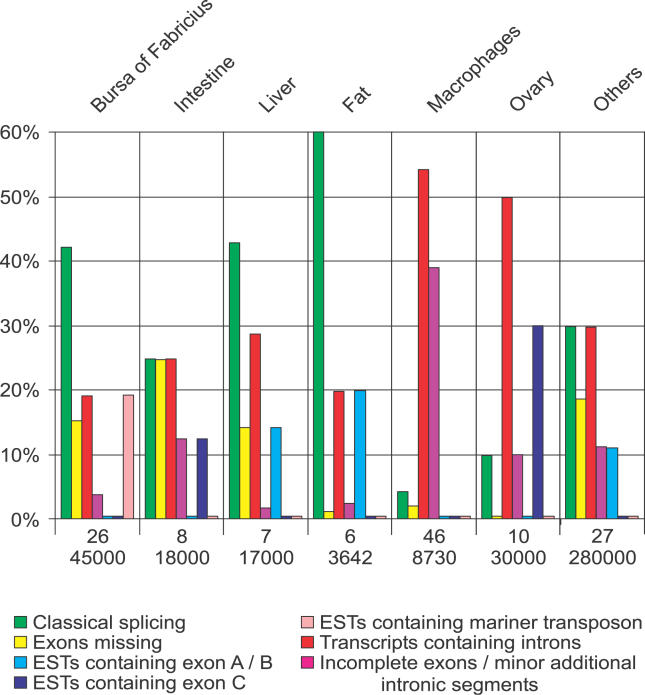
Tissue Distribution of Differentially Spliced CHIR Transcripts The figure gives the relative frequency of a given type of transcript within a tissue. Below the columns, the numbers of CHIR transcripts found and the total number of ESTs available for the respective tissues are specified. Tissues providing less than six CHIR transcripts, as, e.g., kidney or leg muscle, are combined in “others.” This figure represents no tissue specific expression analysis, since ESTs from different genes were subsumed. Additionally, ESTs of most tissues were derived from several different libraries of which some have been normalized, making a direct comparison meaningless [25].

The distribution of differentially spliced variants varies widely in different tissues (Figure 4). Most ESTs derived from the Bursa seem to be functional, while most of the macrophage-derived CHIR ESTs revealed splice variants that do not code for a “conventional” functional receptor protein. Some, however, show proper open reading frames that could lead to nonconventional receptors. The high splicing variability is also chicken strain dependent, since different libraries from the same organ were found to contain variants that differed significantly, e.g., by containing the Bursa alternative exon 7.

Due to the high sequence variability, the assignment of ESTs to their respective CHIR genes was not possible. Phylogenetic trees of exon 3–containing genes and ESTs (not shown) revealed that the transcripts are spread throughout the tree and do therefore not derive from a minor subset of genes. The inability to assign ESTs to their genes, the low abundance of CHIR ESTs in comparison to the high number of loci, and the bias introduced by a mixture of normalized and non-normalized libraries [25] preclude the establishment of expression profiles with the data available.

### Variation Analysis

**Figure 5:**
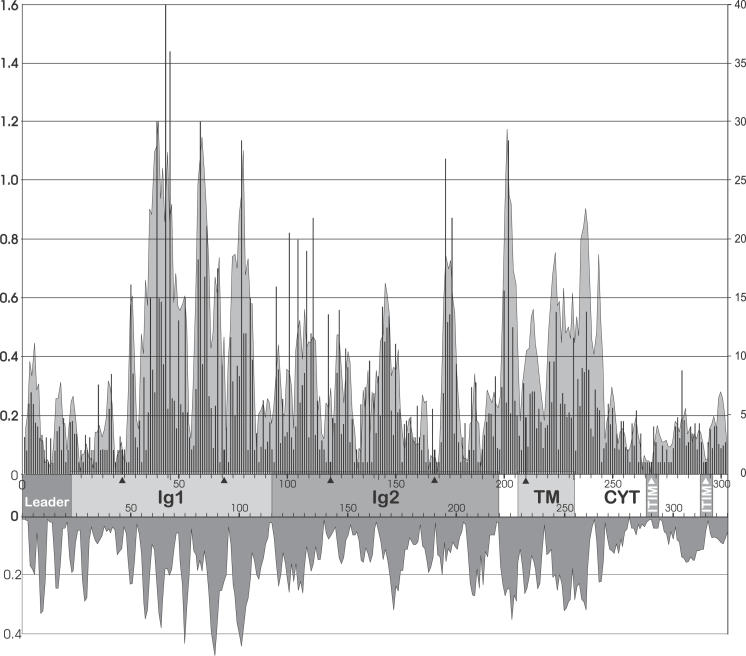
Variability of CHIR Genes The area shaded in light gray in the upper part of the figure demonstrates nonsynonymous exchange rates calculated for each codon and all pairwise comparisons of the 62 potentially functional CHIR genes according to the scale on the left. The overlaid black bars delineate the respective amino acid-based Wu-Kabat [26] values according to the scale on the right. Bars of conserved cysteines are marked with a black arrowhead and bars of ITIM tyrosines are marked with a white arrowhead. The gray area in the lower part of the figure shows the respective synonymous exchanges. Between the two plots, the domain structure and the numbering of amino acids of the immature (bottom) and the mature protein (top) are given.

In addition to the large number of splice variants and their differential tissue expression, the degree of homology between CHIR genes depends on the exon or intron that is examined. While exons 1 and 2 show the highest levels of conservation, homology declines farther downstream. Exon 5 exhibits, in addition to sequence variability, seven size variants and exons 6 and 7 are predicted for about half of the genes only (Figure 2). Complexity is even more pronounced for intronic sequences. Many gene combinations share conservation for introns 1 and 2 but lack significant homology for introns 5 and 6. Analysis of synonymous/nonsynonymous base exchanges resulted in three regions of very high variability for the first Ig domain and in four such regions for the second Ig domain (Figure 5). Moderately elevated synonymous exchange rates within the highly polymorphic regions are an inherent property of the algorithm employed. A variability plot describing the relation between the number of different amino acids at a given position and the corresponding frequency of the most common amino acid [26] supports these findings (Figure 5).

### Phylogenetic Analysis

**Figure 6:**
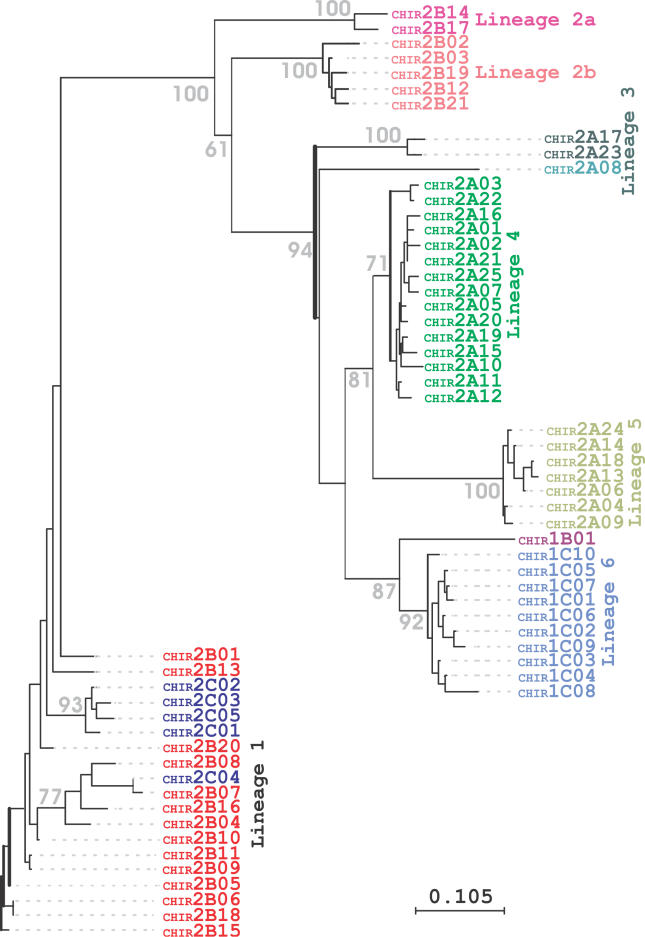
Phylogenetic Tree of CHIR Genes The presented tree was calculated by Phylowin with exon 5 sequences and the following parameters: Neighbor joining with pairwise gap removal, Kimura distance, and 1,000 bootstrap replicates. Bootstrap values are given in gray at the major branches. *CHIR2B15* was used as root, since it clusters with *LILRB2* in the exon 4 tree and no sequences of other species homologous to exon5 could be detected in databases. Further trees with other topographies verified clustering of lineage 1 genes with a bootstrap value of 100 (not shown). Designations of genes were color-coded according to their status: red tones—inhibitory receptors, green tones—activating receptors, and blue tones—bifunctional receptors. To allow easy differentiation between lineages, each lineage was given a different hue. Trees corresponding to exons 2 and 3 are available at http://www.charite.de/immungenetik/CHIR.

To address CHIR gene ancestry, phylogenetic trees of exons 1 to 5 were calculated (see http://www.charite.de/immungenetik/CHIR). However, highly significant results could be obtained only for exon 5, resulting in the arrangement of CHIR genes in six distinct lineages (Figure 6). Additional support for the presented ancestry is provided by lineage-specific 3- and 6-bp deletions in exon 5 (see http://www.charite.de/immungenetik/CHIR alignment) and the shifted position of the arginine within the TM domains of bifunctional CHIR2C versus CHIR2A and CHIR1C receptors. Together with relatively dissimilar neighboring TM sequences, an independent genesis of this positively charged residue is highly likely.

## Discussion

### CHIR Cluster Complexity

Our analysis revealed the LRC of the White Leghorn chicken to be greatly expanded, containing more than 100 CHIR-like sequences that are located on the same microchromosome, which has been identified as 31 in the course of another study [8]. Clone gaps and the lack of identifiable LRC framework loci suggest that even more CHIR genes are present in the LRC of this chicken strain. Despite this uncertainty, our data define this region as one of the most complex Ig receptor gene-containing regions described to date.

The comparison of haplotypes is very valuable for the assessment of the flexibility of a genomic region. However, no indications could be found for an allelic relationship of the BAC contigs to each other and to the sequences determined by the chicken sequencing consortium. In addition, the guided assembly of WGS sequences failed, probably due to high interstrain differences. Since haplotype comparison of distinct individuals by RFLP experiments [8] had already revealed high variability, we conclude that an excessive number of very different LRC haplotypes exists, possibly surpassing the situation of the NITR cluster [27]. The huge differences between CHIR haplotypes combined with the extremely high meiotic recombination rate observed between loci on microchromosomes [24] could result in a newly recombined haplotype after each meiosis and therefore lead to receptors showing exceptional flexibility.

In accordance with the data-release policy of the Sanger Institute, the sequences containing the CHIR genes were immediately submitted to public databases with their status (e.g., finished, unfinished) clearly marked. While finishing was still in progress, Nikolaidis and coworkers [23] published an analysis that contains a number of errors: ten loci containing premature STOP codons or frameshift mutations were incorrectly defined as ordinary CHIR genes (see Table S1) and, in five cases *(CHIR2A6, −18, CHIR2C4, −5,* and *CHIR2B14),* wrong subclasses (activating, inhibitory, or bifunctional) were assigned. Additionally, the phylogenetic trees that were deduced contained duplicates of CHIR genes in different branches *(CHIR2DP4, CHIR2DS16,* and *CHIR2DL17)* (Figure 3) [23], and an undefined locus (*CHIR2DL25*). Consequently, the resulting phylogenetic analysis must be treated with caution.

### CHIR Cluster Expansion

An interesting feature of the chicken LRC concerns the presence of genomic blocks containing two CHIR genes and an intergenic region that together seem to constitute the basic duplication unit (Figure 2). This unit occurs in three versions: (i) two activating receptors (Figure 2, red block); (ii) an activating and an inactivating receptor (Figure 2, green block); and (iii) a bifunctional and an inactivating receptor (Figure 2, yellow block). Although some of the resulting CHIR copies are not functional in the haplotype analyzed here, it is tempting to speculate that activating and inhibitory receptor pairs are functionally interrelated, such as *Klra8* and *Klra9* in mice [20]. In addition to these large scale duplications, single gene duplications probably also occurred, since a number of genes were identified in a context not found elsewhere (e.g., *CHIR2B13* and *CHIR2B4*). In agreement with the phylogenetic results, exon shuffling or other intergenic rearrangements have probably been rare events in CHIR evolution, in marked contrast to the domain shuffling mechanism found for KIR [15] or Klra genes [13].

### Diversification of the Putative Ligand Binding Interface of CHIRs

The high level of diversification is not only a feature of CHIR genes but is shared by many other receptor families of vertebrate innate immune systems like LILR, KIR, Pir, NITR, SIRP, and Klra [27–30]. It facilitates the optimization of different ligand specificities for individual members of the receptor family and is often a reflection of protein function. We were therefore interested to determine whether selection is involved in the fixation of the observed variation. Variability plots comparing the different CHIR genes (Figure 5) revealed the presence of hypervariable regions with clear preponderance of nonsynonymous substitutions. The substitution rate within and around the TM domain, probably due to diminished structural constraints, is also relatively high, fitting well the observation that no significant similarity could be detected with any known nonchicken gene.

To investigate whether these hypervariable regions might be involved in ligand binding, the respective residues were mapped onto the surface of LILRB1 and KIR2DL2/HLA class I structural models [31,32], and indeed, most of the hypervariable residues are predicted to be located close to the putative ligand/receptor interface (Figure S1). Since all known ligands of the LILR and KIR protein families are in the MHC protein family, it is conceivable that CHIRs interact with the BF (MHC class I) molecules of the chicken [33–35] and/or with the MHC class I–like Y antigens, which not only exhibit a limited degree of polymorphism [36,37] but occur also in variable number depending on the chicken strain [34,37]. However, the role of highly variable amino acids might have to be questioned when an LILR-like MHC binding mode [32] is assumed for the CHIRs [8], since the hypervariable regions would be predicted to contact β2m or the α3 domain of an MHC class I heavy chain, both of which are highly conserved. In contrast, several positions of the hypervariable residues are supportive of the KIR model (Figure S1): residues 44 and 45 (corresponding to CHIR positions 40 and 41) of activating two-domain KIRs control discrimination between the C1 and C2 HLA-C allotypes [38], KIR2DL2 residues 71 and, to a weaker extent, 104 (CHIR positions 63 and 95, respectively) are involved in peptide recognition [31], and residues 67 to 70 of KIR2DS1 and KIR2DS2 (CHIR positions 59 to 62) are critical for contact of these activating KIRs with HLA-C molecules [38,39]. Yet, the existence of CHIRs without Ig domain 2 indicates that further ligands, probably exhibiting a different binding mode including the potential for homotypic CHIR/CHIR interactions demonstrated for surface proteins encoded in the natural killer cell complex of mice [40,41], may exist. Whatever the nature of CHIR ligands will turn out to be, the enormous expansion of CHIR genes combined with tremendous diversification of the putative binding interface supports the hypothesis that the conventional distinction between receptors of adaptive and innate immunity is blurred in certain species [42]. We would like to point out, however, that not only the differences between individual CHIR genes could be relevant for ligand recognition but also allelic polymorphisms. However, this additional level of variability is currently supported only indirectly, by the failure to assign ESTs and cDNAs to distinct CHIR genes of the White Leghorn chicken.

### Transcript Variability

According to the results described above, CHIR sequences seem to be subject to selection, especially in the hypervariable regions, to optimize the ligand binding properties. However, the effects of nucleotide exchanges are not necessarily limited to amino acid exchanges. Altered splice sites generate novel transcripts which may be functional as described for KIRs [43], but many of these splice site alterations might simply be a consequence of rapid evolution. Some cell types, like macrophages, gave predominantly rise to such “non-functional” splice variants, and some of these variants were found solely in a single library. Since cDNA libraries from different tissues are usually generated from independent animals, this variability could be a result of the analysis of distinct LRC haplotypes. In addition, diversification seems to affect the promoter of the respective genes: the rate of “correctly” spliced CHIRs is maximal for Bursa ESTs, while distinct CHIR transcripts were not detectable in Bursa RNA by RT-PCR in a different animal [8]. Alternative promoters may also operate for transcripts starting with exons A and B (Figure 4), which were found so far only in tissues lacking a primary immune function. In summary, transcript variability is very high at all levels and parallels the pronounced genomic flexibility.

### Phylogeny

**Figure 7:**
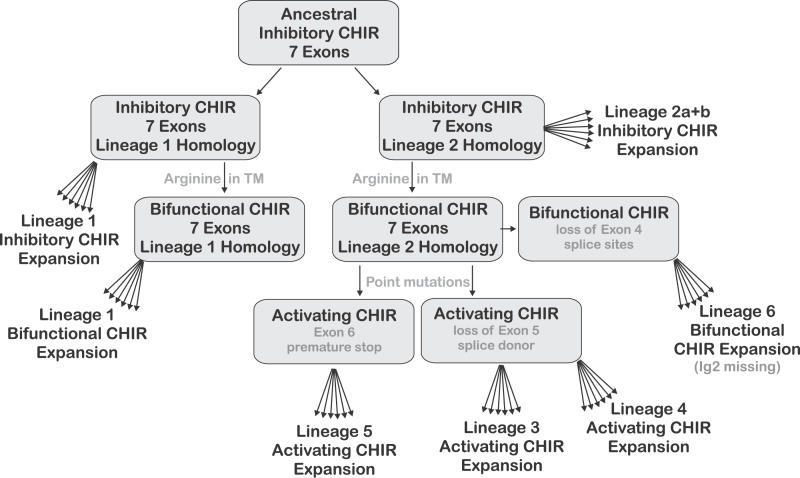
Suggested Evolution of CHIR Genes This hypothetical evolutionary tree for CHIR genes is largely based on phylogenetic analysis of exon 5. Evolutionary intermediates are boxed; lineage designations are according to Figure 6.

The analysis of CHIR ancestry was carried out by phylogenetic analysis (Figure 6). Most informative were exon 5 trees that facilitated the definition of clear-cut lineages and led to the development of the evolutionary model presented in Figure 7: Significant homology between the ITIM motive-containing domains of inhibitory CHIR, LILR and Pir genes, as well as the corresponding pseudoexons 6 and 7 that are still present in many CHIR2A genes show that the primordial receptor must have been inhibitory. Such a primordial receptor delivering an inhibitory signal upon detection of its ligand has been hypothesized to have existed very early in vertebrate development [19,44,45]. At least in the ancestors of White Leghorn chickens, the development of activating variants from the chicken inhibitory receptor happened twice (Figure 7). In contrast, all activating hominoid KIR known to date result from only one such event, and a comparable situation was observed for the activating old world monkey KIRs and the rodent Klra genes [13]. Three independent modifications led to loss of the ITIM-containing parts of the cytoplasmic domain in the three CHIR2A lineages, while the CHIR1C and CHIR2C lineages retained the respective motives. In this context, it is also noteworthy that more activating (25) than inactivating (22) receptors were detected among CHIRs and the 15 remaining bifunctional genes code for receptors that are potentially activating, like e.g., *KIR2DL4* [46,47]. This suggests a potential evolutionary advantage connected with the development of activating CHIRs.

### Conclusions

The extraordinarily high number of CHIR genes that evolve very fast provides a broad spectrum of immune receptors for a given bird. In addition, the dramatic differences between LRC haplotypes expand the versatility of CHIRs for the whole species. Together with the expression in different cell lineages, it is conceivable that this highly variable receptor family serves the innate as well as the adaptive branch of the chicken's immune system. This could be a compensation for the reduced number of MHC class I genes in chicken [33,35,48] and the ensuing reduction in T cell diversity that is probably a consequence [49]. For example, in sticklebacks *(Gasterosteus aculeatus),* the capacity to mount adequate adaptive immune responses depends on an optimal number of MHC alleles. When this optimum is not reached in an animal, innate immune responses appear to be upregulated [50]. Whether the complexity of the chicken's LRC together with a “minimal essential MHC” [33] is just another solution to fend off pathogens or part of a hitherto unobserved strategy to reduce the high price in terms of autoimmunity and hypersensitivity attributable to a highly sophisticated adaptive immune system [51] awaits further analysis.

## Materials and Methods

### Library screening.

The “Chicken Genomic BAC Library” was made from blood cells of a single, noninbred female White Leghorn chicken [52] and purchased at the MRC Gene Service (Cambridge, United Kingdom), while a chicken bursa of Fabricius cDNA macroarray made from purified B cells of a single female from the inbred CB line [53] was purchased from RZPD (Berlin, Germany). Both were screened by hybridization with the probes “CHIR-A” and “CHIR-B” (for details, see below). Probe labeling, hybridization, and clone detection were done as described previously [54]. BAC signals were unusually strong and, despite low stringency conditions, almost no background was observed even after prolonged exposure. Following recloning, BAC clones were cultured in LB with chloramphenicol (12.5 μg/ml) and, finally, DNA was isolated by a standard alkaline lysis procedure. Rescreening of the EcoRI-digested BAC DNA with the probes “CHIR-A” and “CHIR-B” resulted in numerous CHIR-positive bands for all clones. Using restriction fingerprinting [55,56], BACs were mapped into contigs. The BAC library macroarrays and zoo-blots were further hybridized with probes for the following LRC framework loci: *NKp46* (bovine) and human *LILRA2, NCR1, FCAR,* as well as a KIR mix *(KIR2DL1, KIR2DL3, KIR3DL1,* and *KIR3DL2)*.

### FISH.

Metaphase and interphase chromosome preparations were obtained from short-term culture of primary fibroblasts of a Rhode Island Red chicken embryo (9-day egg). The cells were cultured in Earle's medium 199 (supplemented with 10% newborn bovine serum and tryptose phosphate broth) until confluency. They were then treated with hypotonic solution (0.0075 M KCl) for 20 min at 37 °C and fixed in three changes of Carnoy fixative (3:1 methanol acetic acid). Slides were prepared following standard procedures and aged at −20 °C.

BAC probes were labeled by nick-translation (Vysis kit) either with biotin-16-dUTP or digoxigenin-11-dUTP (Roche, Basel, Switzerland). Following labeling, they were ethanol precipitated in a mix of Salmon testis DNA (GIBCO-BRL, San Diego, California, United States), Escherichia coli tRNA (Boehringer, Ingelheim, Germany), and 3M sodium acetate. They were then dried on a heating block at 60 °C, resuspended at 20 ng/μl in hybridization solution (50% formamide, 10% dextran sulfate, 2× SSC), and denatured at 80 °C for 5 min before being applied to the slide under a 22 × 22-mm coverslip. Prior to hybridization, the slides were denatured in 70% formamide at 70 °C for 1.5 min, quenched in cold 2× SSC, and dehydrated in an ethanol series. After being checked one by one in single-color hybridizations, the probes were combined in double-color FISH experiments for colocalization assessment.

Following hybridization in a moist chamber at 37 °C for 20 h, the slides were washed in 50% formamide at 42 °C for 10 min and 2× SSC at 42 °C for 5 min. Biotinylated probes were detected with Texas Red–conjugated streptavidin (Molecular Probes, Eugene, Oregon, United States). Probes labeled with digoxigenin were detected using mouse anti-digoxigenin antibody (Roche) and goat anti-mouse Alexa-488 (Molecular Probes). The slides were mounted with Vectashield (Vector Laboratories) containing 4′,6-diamidino-2-phenylindole (DAPI) for chromosome counterstaining.

The slides were examined using an Olympus BX-51 epifluorescence microscope coupled to a Sensys charge-coupled device (CCD) camera (Photometrics). A minimum of 25 metaphases were analyzed for each hybridization experiment. Texas Red, Alexa-488, and DAPI fluorescence images were taken as separate gray-scale images using specific filter combinations and then pseudocolored and merged using the software package Genus (Applied Imaging International, Newcastle upon Tyne, England). Fiber-FISH images were captured and analyzed using the software package Metamorph (Universal Imaging Corporation, Downingtown, Pennsylvania, United States).

### Sequence determination and analysis.

The BACs were sequenced at The Wellcome Trust Sanger Institute and the corresponding sequences were submitted to the EMBL/Genbank/DDBJ databases. Published exon sequences of CHIR-A (five exons) and CHIR-B (seven exons) [19] and ESTs with CHIR homology were used to detect CHIR-similar sequences in the BACs. Since similarity downstream of exon 4 is often low, multiple exon/intron sequences of different CHIR genes/cDNAs were used in parallel for the similarity search. For the search of exon A–/intron A–homologous sequences, the genomic configuration found for *CHIR2A14* was used as model. Splice donors and acceptors were verified manually. Alignments of low homology CHIR gene regions were carried out with MultAlin (http://prodes.toulouse.inra.fr/multalin/multalin.html). Protein feature prediction was performed with “SMART” (http://smart.embl-heidelberg.de), and the repeat masker program (A. F. A. Smit and P. Green, unpublished data, http://www.repeatmasker.org) was employed to search for repetitive elements. For the assignment of intergenic regions, BAC sequences were masked for CHIR genes and repetitive elements, and then compared to each other by dot matrix analysis [57]. Four intergenic regions were defined between *CHIR2A2* and *CHIRP1, CHIRP8* and *CHIR2A5, CHIRP25* and *CHIR2B10,* as well as *CHIR2B18* and *CHIR2A25*. Homology of these regions with the human LRC genes *NKp46, FCAR, LENG8,* and *RPS9* [5] has been excluded by dot matrix analysis. Wu-Kabat values were calculated by an in-house programmed Excel script with the following formula:


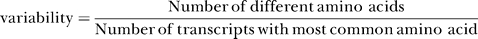


### WGS.

The original reads of the WGS were accessed using the trace archive (http://trace.ensembl.org/perl/ssahaview?server=gallus_gallus and http://www.ncbi.nlm.nih.gov/blast/mmtrace.shtml), revealing 316 hits with nearly exact match. Relaxing the matching criterion, we could still find a good portion of the reads which could be assigned to the local region. A manually assisted assembly of selected clones was first tested using local clone reads as well as WGS reads with a good match to the clone sequences. The trial failed as the region is repetitive. A second strategy was pursued to produce an alternative assembly, using BAC sequences as a framework. BAC clone sequences were shredded into pieces with a read length of 1,000 bp and a paired insert size of 4,000 bp. The shredded reads accounted for about 5× coverage over the BACs. A total number of 12 million WGS reads of Red Jungle Fowl chicken were downloaded from the Ensembl trace repository (ftp://ftp.ensembl.org/pub/traces/gallus_gallus). The Phusion assembler [58] was used to assemble the genome. It was hoped that we could close gaps between BAC clone contigs or perhaps new assembly sequences could extend into the neighboring LRC regions if there were enough WGS reads in the region. However, it seemed to be the case that for the WGS reads there was lack of coverage in the LRC region, judging from the Phusion assembly. BAC clone sequences were found to match the contigs in the new assembly 100% and by examining the read placement file, very few WGS reads were placed in the region. Within one supercontig which covers the CHIR cluster, we found two contigs (13 and 57 kb) without homology to the BACs presented here. The Phusion assembly is available at ftp://ftp.sanger.ac.uk/pub/zn1/chicken.

### Probe generation.

Hybridization probes were synthesized from 5 μg of peripheral blood RNA of noninbred female White Leghorn chicken, using the “PolyT-Smart” primer (gaagcagtggtaacaacgcagagtactTTTTTTTTTTTTTTTTTTTTTVN) and 200 U of reverse transkriptase (Superscript II). PCRs were performed with the following primer combinations: primer CHIR_all_Fw_01 (GCGTCCGCTCGCTGTC)/CHIR_A_Rv (GTGGGCAAGAGATTAAATGGCTTT [19]): “CHIR-A” probe, CHIR_all_Fw_01/CHIR_B_Rv (ACAGTGCCAGTGTGCTCGGTGTGCTCA [19]): “CHIR-B” probe, CHIR_all_Fw_02 (TGGCACCAATGGCAGTGGC)/SMART_Tag_1 (GAAGCAGTGGTAACAACGCAGA). PCR conditions were 2 min at 94 °C; 35 cycles of 20 s at 94 °C, 15 s at 63 °C, 90 s at 68 °C; and 7 min at 68 °C. After TOPO-TA-cloning (Invitrogen), the identity of the CHIR inserts was checked by sequencing.

### EST analysis.

ESTs with CHIR homology were collected from the following databases: 19 ESTs from UMIST [59] and 119 ESTs from dbEST (http://www.ncbi.nlm.nih.gov). Several alignment programs (e.g., ClustalW [60], Multalign [61], and Chaos+Dialign [62]) were employed to analyze the sequences, but it became clear very soon that the respective algorithms are not suitable for this complex problem. Two major points are responsible for the failure: (i) transcripts revealed many different splice variants and (ii) the CHIR genes are very polymorphic. It was therefore necessary to apply a semimanual strategy, which is based on pairwise alignments between single CHIR-exons and the expressed sequences of interest. The final alignment is available at http://www.charite.de/immungenetik/CHIR.

### Phylogenetic analysis.

Phylogenetic trees from exons 1 + 2, 3, 4, and 5 were calculated using Phylowin [63] and checked for exon 3 + 5 with PHYLIP (J. Felsenstein, 2004, Phylogeny Inference Package version 3.6., Department of Genome Sciences, University of Washington, Seattle, Washington, United States) resulting in virtually the same results. Parameters are given in Figure 6. This analysis was validated by the compilation of additional tree topographies and by tree construction with the maximum parsimony algorithm (unpublished data). Furthermore, trees including CHIR sequences from the WGS and from EST libraries were also calculated without significant changes to lineage classification of the CHIRs described here.

## Supporting Information

Figure S1 Predicted Structures of CHIR2C2 The upper panel shows the surfaces of the human LILRB1 and the respective CHIR2C2 model. In the lower panel, KIR2DL2 and the respective CHIR2C2 model are shown. To improve orientation, binding interfaces of HLA class I heavy chain and β2m as well as a peptide presented by the HLA class I antigen (lower panel only) are indicated. The front of the respective receptor is that known or proposed to interact with an MHC molecule. Amino acids of the CHIR2C2 models were colored according to the Wu-Kabat variability plot (Figure 5). Numbering of residues within the mature protein is given only for residues with a Wu-Kabat value greater than 20 (in the KIR model, positions 59 and 62 were labeled additionally). Models were calculated by homology modeling using DeepView for alignment (http://swissmodel.expasy.org) and the protein modeling server SWISS-MODEL. The following structures were used as templates: LILR model and KIR model. Illustrations of color-coded surfaces were generated with PYMOL (http://www.pymol.org).
(2.2 MB JPG)

Table S1 Summary of Genomic Structure and Defects of CHIR Pseudogenes (84 KB DOC)

### Accession Numbers

BAC sequences are available from the EMBL/GenBank/DDBJ databases (http://www.ebi.ac.uk/embl/index.html) under the accession numbers BX663523, BX663526, BX663527, BX663529, BX663530, BX663534, and BX897752.

The National Center for Biotechnology Information (NCBI) (http://www.ncbi.nlm.nih.gov) accession numbers for the genes referred to in this publication are *NCR1* (9437); *NCR2* (9436), *LILRA2* (11027), *CHIRA* (AF306851) and *CHIRB* (AF306852), *NKp46* bovine (369024), *FCAR* (2204), *KIR2DL1* (3802), *KIR2DL3* (3804), *KIR2DL4* (3805), *KIR3DL1* (3811), and *KIR3DL2* (3812).

The Protein Data Bank (http://www.rcsb.org/pdb) accession numbers for structures used as templates are LILR model (1g0xA, 1ugnA, 1p7qD, and 1ufuA) and KIR model (1im9D and 1efxE).

## Abbreviations

CHIR: chicken immunoglobulin-like receptor
EST: expressed sequence tag
FISH: fluorescent in situ hybridization
Ig: immunoglobulin
ITAM: immune receptor tyrosine-based activatory motif
ITIM: immune receptor tyrosine-based inhibitory motif
ITSM: immune receptor tyrosine-based switch motif
KIR: killer cell Ig-like receptor
Klra: killer cell lectin-like receptor subfamily A
LILR: leukocyte immunoglobulin-like receptor
LRC: leukocyte receptor complex
MHC: major histocompatibility complex
Pir: paired Ig-like receptor
TM: transmembrane
WGS: whole genome shotgun
