# Supplementary material for: The Leukocyte Receptor Complex in Chicken Is Characterized by Massive Expansion and Diversification of Immunoglobulin-Like Loci
Source: PLoS Genet. 2006 May 12;2(5):e73. doi: 10.1371/journal.pgen.0020073 (PMC1458963; doi:10.1371/journal.pgen.0020073)
Supplement: Supplementary file 1 [file pgen.0020073.st001.doc]

| Name | Exon 1 | Exon 2 | Exon 3 | Exon 4 | Exon 5 | Exon 6 | Exon 7 |
| --- | --- | --- | --- | --- | --- | --- | --- |
| ***CHIRP4 112A23_Gen09*** | 🡪 + 🡨 | 🡪 + 🡨 | 🡪 +S🡨 | 🡪 + 🡨 | 🡪 + 🡨 | 🡪 + 🡨 | 🡪 + 🡨 |
| ***CHIRP32 19H9_Gen14*** | 🡪 + 🡨 | 🡪 + 🡨 | 🡪 +S🡨 | 🡪 + 🡨 | 🡪 + 🡨 | 🡪 + 🡨 | 🡪 + 🡨 |
| ***CHIRP18 52G8_Gen17*** | 🡪 + 🡨 | 🡪 + 🡨 | 🡪 + 🡨 | 🡪 +S🡨 | 🡪 + 🡨 | 🡪 + 🡨 | 🡪 + 🡨 |
| ***CHIRP24 52G8_Gen24*** | 🡪 + 🡨 | 🡪 + 🡨 | 🡪 + 🡨 | 🡪 +S🡨 | +F | 🡪 + 🡨 | 🡪 + 🡨 |
| ***CHIRP9 112A23_Gen18*** | 🡪 + 🡨 | 🡪 + 🡨 | 🡪 + 🡨 | 🡪 +F🡨 | 🡪 + 🡨 | 🡪 + 🡨 | 🡪 + 🡨 |
| ***CHIRP43 4C11_Gen12*** | 🡪 + 🡨 | 🡪 + 🡨 | 🡪 +S🡨 | 🡪 +S🡨 | 🡪 + 🡨 | + | - |
| ***CHIRP21 52G8_Gen21*** | 🡪 + 🡨 | 🡪 + 🡨 | 🡪 + 🡨 | 🡪 +FS🡨 | 🡪 + 🡨 | 🡪 + 🡨 | 🡪 + 🡨 |
| ***CHIRP10 58B13_Gen05*** | 🡪 + 🡨 | 🡪 + 🡨 | 🡪 + 🡨 | 🡪 +FS🡨 | 🡪 + 🡨 | 🡪 + 🡨 | - |
| ***CHIRP25 19H9_Gen01*** | 🡪 + 🡨 | 🡪 + 🡨 | 🡪 + 🡨 | + | 🡪 + 🡨 | - | 🡪 + 🡨 |
| CHIRP30 19H9_Gen12 | 🡪 + 🡨 | 🡪 + 🡨 | 🡪 +S🡨 | 🡪 + 🡨 | 🡪 + 🡨 | - | + |
| ***CHIRP1 112A23_Gen04*** | 🡪 + 🡨 | 🡪 + 🡨 | 🡪 + 🡨 | 🡪 +S🡨 | 🡪 + 🡨 | - | - |
| CHIRP17 52G8_Gen14 | 🡪 + 🡨 | 🡪 + 🡨 | 🡪 +S🡨 | 🡪 + 🡨 | 🡪 + 🡨 | - | - |
| CHIRP14 52G8_Gen06 | 🡪 + 🡨 | 🡪 + 🡨 | 🡪 + 🡨 | 🡪 +S🡨 | + | - | - |
| CHIRP6 112A23_Gen11 | 🡪 + 🡨 | 🡪+FS🡨 | 🡪 + 🡨 | - | - | - | - |
| CHIRP8 112A23_Gen15 | 🡪 + 🡨 | 🡪 + 🡨 | 🡪+FS🡨 | 🡪 + 🡨 | - | - | - |
| CHIRP38 4C11_Gen02 | 🡪 + 🡨 | 🡪 + 🡨 | 🡪 +S🡨 | 🡪 + 🡨 | - | - | - |
| CHIRP3 112A23_Gen07 | 🡪 + 🡨 | 🡪 + 🡨 | 🡪 + 🡨 | + | - | - | - |
| CHIRP7 112A23_Gen13 | 🡪 + 🡨 | 🡪 + 🡨 | 🡪 + 🡨 | + | - | - | - |
| CHIRP19 52G8_Gen18 | 🡪 + 🡨 | 🡪 + 🡨 | 🡪 +S🡨 | + | - | - | - |
| CHIRP29 19H9_Gen09 | 🡪 + 🡨 | 🡪 + 🡨 | 🡪 + 🡨 | - | + | - | + |
| CHIRP11 58B13_Gen06 | 🡪 + 🡨 | 🡪 + 🡨 | 🡪 + 🡨 | - | - | - | + |
| CHIRP12 58B13_Gen09 | 🡪 + 🡨 | 🡪 + 🡨 | 🡪 + 🡨 | - | - | - | - |
| CHIRP13 52G8_Gen02 | 🡪 + 🡨 | 🡪 + 🡨 | 🡪 + 🡨 | - | - | - | - |
| CHIRP35 **93H17_Gen03**  ehem. Gen08 | 🡪 + 🡨 | 🡪 + 🡨 | 🡪 +S🡨 | - | - | - | - |
| CHIRP23 52G8_Gen23 | 🡪 + 🡨 | 🡪 + 🡨 | 🡪 + 🡨 | - | - | - | - |
| CHIRP16 52G8_Gen11 | 🡪 + 🡨 | 🡪 + 🡨 | 🡪 + 🡨 | - | - | - | - |
| CHIRP22 52G8_Gen22 | 🡪 + 🡨 | 🡪 + 🡨 | 🡪 + 🡨 | - | - | - | - |
| CHIRP39 4C11_Gen05 | 🡪 + 🡨 | 🡪 + 🡨 | 🡪 + 🡨 | - | - | - | - |
| CHIRP42 4C11_Gen11 | 🡪 + 🡨 | 🡪 + 🡨 | 🡪 + 🡨 | - | - | - | - |
| CHIRP40 4C11_Gen09 | 🡪 + 🡨 | + | 🡪 + 🡨 | - | - | - | - |
| CHIRP2 112A23_Gen06 | + | - | - | + | - | - | - |
| CHIRP20 52G8_Gen19 | - | - | + | - | - | - | - |
| CHIRP36 **93H17_Gen04**  ehem. Gen09 | - | - | + | - | - | - | - |
| CHIRP41 4C11_Gen10 | - | - | + | - | - | - | - |
| CHIRP5 112A23_Gen10 | - | - | - | 🡪 + 🡨 | - | - | - |
| CHIRP26 19H9_Gen02 | - | - | - | + | - | - | - |
| CHIRP28 19H9_Gen08 | - | - | - | + | - | - | - |
| CHIRP31 19H9_Gen13 | - | - | - | + | - | - | - |
| CHIRP33 19H9_Gen18 | - | - | - | - | - | - | + |
| CHIRP34 19H9_Gen20 | - | - | - | - | - | - | + |
| CHIRP15 52G8_Gen09 | - | - | - | - | - | - | + |

Summary of genomic structure and defects of CHIR pseudogenes.

Delineated are the defects turning the respective sequence into a pseudogene as well as the elements that look still functional. Pseudogenes are ordered according to increasing stages of decay. Symbols of pseudogenes that were given a functional status previously (Nikolaidis et al. 2005) were italicized.

Arrows: Splice acceptor (🡪) / donor (🡨)

+	Homology to the respective exon or parts of it

S	premature STOP codon in this exon

F	frame shift
